# Draft genome sequence of *Stenotrophomonas maltophilia* strain TUM26315, a bloodstream isolate resistant to cefiderocol and to aztreonam combined with ceftazidime-avibactam

**DOI:** 10.1128/mra.00949-25

**Published:** 2025-10-30

**Authors:** Takashi Sakoh, Kohji Komori, Sohei Harada, Kageto Yamada, Hideki Araoka

**Affiliations:** 1 Department of Infectious Diseases, Toranomon Hospital 13600, Minato-ku, Tokyo, Japan; 2 Okinaka Memorial Institute for Medical Research 545161, Minato-ku, Tokyo, Japan; 3 Division of Collaborative Regional Infection Control, Department of Community Well-being, Toho University School of Medicine 36591 , Ota-ku, Tokyo, Japan; 4 Department of Microbiology and Infectious Diseases, Toho University School of Medicine 36591 , Ota-ku, Tokyo, Japan; University of Pittsburgh School of Medicine, Pittsburgh, Pennsylvania, USA

**Keywords:** *Stenotrophomonas maltophilia*, cefiderocol, resistance, *cirA*

## Abstract

Here, we report the draft genome sequence of *Stenotrophomonas maltophilia* TUM26315, which exhibits resistance to cefiderocol and to aztreonam combined with ceftazidime-avibactam, despite no prior exposure. The isolate belonged to genomic group 6 and carried *bla*_L1B_ and *bla*_L2B _β-lactamase genes. It also had a frameshift mutation in the *cirA* gene.

## ANNOUNCEMENT

 Cefiderocol has shown activity against *Stenotrophomonas maltophilia,* with resistance being rare in clinical isolates (1–4). In our previous study, only one isolate among 146 *S*. *maltophilia* bloodstream isolates collected in Japan before the approval of cefiderocol and ceftazidime-avibactam was resistant (MIC ≥ 64 µg/mL) to cefiderocol (5). We performed whole-genome sequencing of the cefiderocol-resistant strain, designated TUM26315. This study, which involves only microbiological and genetic analysis of clinically isolated strains, does not require informed consent under the Japanese Guidance on Ethical Guidelines for Life Science and Medical Research Involving Human Subjects.

  The original strain was recovered from aerobic blood culture using the BD BACTEC FX system (Becton Dickinson, Sparks, MD, USA) and isolated on 5% sheep blood agar at 35°C under 5% CO_2_. Bacterial DNA was extracted from the strain grown on Mueller-Hinton agar at 35°C under aerobic conditions using magLEAD 6gC and MagDEA Dx SV with PS protocol (Precision. System Science Co., Ltd., Chiba, Japan). DNA libraries were prepared using Illumina DNA Prep (Illumina, San Diego, CA, USA) and sequenced on the MiSeq platform with 300 bp paired-end reads. The raw reads were trimmed using Trimmomatic version 0.39 (quality score cutoff, 30) (6) and assembled with SPAdes version 3.15.4 (7). Annotation was performed using the DFAST pipeline version 1.3.6 (8). The draft genome consists of 105 contigs, totaling 4,482,155 bp, with an *N*_50_ value of 85,448 bp and 201,099,535 raw nucleotides, achieving 45× coverage. Annotation predicted 4,030 coding sequences and 73 tRNA genes. The GC content was 66.7%. The strain was genomically identified as *S. maltophilia* by average nucleotide identity analysis using the DFAST_QC pipeline version 1.0.7 (9), showing 98.16% identity to the genome of *S. maltophilia* NBRC 14161^T^ and belonging to genomic group 6 (10, 11).

 The nucleotide sequences of the genes previously implicated in the development of cefiderocol resistance, *tonB*, *exbD*, *smlt1148*, *cirA*, *tolQ*, *smf-1*, and *smeT* (12–14), in TUM26315 were compared with those of the cefiderocol-susceptible strain ATCC 13637 (GenBank accession: CP008838) using nucleotide BLAST version 2.16.0+ (15) (Table 1). A compound frameshift mutation (28_29insGC, 36_37insT, and 61_62insT) that caused a premature stop codon (Asp213X) was documented in the *cirA* gene encoding the catechol-acid siderophore receptor. Absence of the *smf-1* gene, encoding a putative fimbrial adhesin protein precursor, and a mutation in *smeT*, encoding the repressor of multidrug efflux pump SmeDEF, were also documented.

**TABLE 1:** Characteristics of *Stenotrophomonas maltophilia* strain TUM26315

Strain		TUM26315
Minimum inhibitory concentration, µg/mL	Cefiderocol	>32
	Aztreonam	>128
	Ceftazidime-avibactam	>128/4
	Aztreonam/ceftazidime-avibactam	64/8/4
Genes associated with resistance to cefiderocol^*a*^	*tonB*	Wild type
	*exbD*	Wild type
	*tolQ*	Wild type
	*smf-1*	Not detected
	*smeT*	Glu8Asp
	*smlt1148*	Wild type
	*cirA*	Frameshift (insGC, insT×2) causing stop at Asp213

^*a*^ The following gene sequences were obtained from the EMBL database: *tonB* (CAQ43627), *exbD *(CAQ46197), *tolQ *(CAQ47122), *smf-1 *(CAQ44285), *smeT *(CAQ47464), *smlt1148 *(CAQ44704), and *cirA *(CAQ47526). As a reference strain, cefiderocol-susceptible strain ATCC 13637 (GenBank accession: CP008838) was used.

 Additional antimicrobial susceptibility testing using the broth microdilution method revealed that the MIC of aztreonam was 64 µg/mL when tested with a fixed concentration of ceftazidime 8 µg/mL and avibactam 4 µg/mL, suggesting reduced susceptibility to this combination (Table 1). This strain had sequences that were completely identical to *bla*_L1B_ and *bla*_L2B_ (16). In a previous study, 10 of 59 genomic group 6 strains collected in the United States carried *bla*_L1B_ and *bla*_L2B_, and all of these strains, except one that showed intermediate susceptibility, were susceptible to this combination (10). Therefore, the presence of these β-lactamase gene variants is unlikely to be the primary cause of the resistance in this strain.

## Data Availability

The genome sequence of *S. maltophilia* strain TUM26315 is deposited under GenBank accession number JBQPHK000000000.
